# Coherent Fiber-Optic Sensor for Ultra-Acoustic Crack Emissions

**DOI:** 10.3390/s21144674

**Published:** 2021-07-08

**Authors:** Ilaria Di Luch, Maddalena Ferrario, Davide Fumagalli, Michele Carboni, Mario Martinelli

**Affiliations:** 1 Dipartimento di Elettronica, Informazione e Biomedica, Politecnico di Milano, 20133 Milano, Italy; mario.martinelli@polimi.it; 2 Cohaerentia s.r.l., 20133 Milano, Italy; maddalena.ferrario@cohaerentia.com; 3 Dipartimento di Meccanica, Politecnico di Milano, 20156 Milano, Italy; davide11.fumagalli@mail.polimi.it (D.F.); michele.carboni@polimi.it (M.C.)

**Keywords:** fiber optic sensor, guided waves, acoustic emission, fatigue crack propagation

## Abstract

A coherent optical fiber sensor with adequate sensitivity for detecting the acoustic emission (AE) during the propagation of a crack in a ferrous material is presented. The proposed fiber optic sensor is successfully compared in terms of the SNR (Signal to Noise Ratio) and detectable AE energy levels to commercially available AE piezo-transducers sensors and is proven to be an effective and advantageous alternative for sensing and monitoring fatigue damage in structural applications.

## 1. Introduction

Acoustic Emissions (AE) are an established real-time structural health monitoring approach for detecting, localizing, and monitoring damage initiation and development in many structural applications, for example in the mechanical, civil, automotive and aerospace fields made of different technical materials, such as metals, polymers, ceramics and composites. The onset and development of damaging events, such as cracks, delamination, corrosion, and debonding, release energy in terms of transient high-frequency elastic (ultrasonic) waves, which travel into the involved materials assuming the shape of either spherical volumetric waves or guided waves depending on the given boundary conditions.

By acquiring and suitably analyzing such sound waves, it is possible to detect and locate faults in complex structures, exactly as it happens in seismology from which the industrial application of AE was directly derived, even if adapted to a different scale. AE sensors based on piezoelectric transducers (PZT) are the most widespread sensor types currently adopted, featuring high-sensitivity and suitable bandwidth [1,2]. However, PZT transducers work with electric charges, which are low in intensity, and thus an electronic circuitry is needed close to the sensor transducer (e.g., a couple of meters) in order to avoid severe propagation losses as well as bandwidth limitations.

Moreover, the connecting cable must be electrically shielded in order to avoid electro-magnetic noise (e.g., a coaxial cable), and the results are very sensitive to environmental perturbation (e.g., vibrations and acoustic noise) [3]. These “interface” limitations have shrunk the deployment of PZT sensors in applications that require a remote measurement, such as field applications.

In such a context, fiber-optic sensors, FOS, have been investigated through the years as a possible alternative. The greatest advantages of FOS are the total electromagnetic immunity, the possibility to easily have the interrogation unit remote from the transducer unit, the capability of withstanding harsh environments, and the availability of many multiplexing options (time, space, as well as wavelength). In front to these recognized advantages, it is not yet well established if FOS offer the same high-sensitiviy of PZT transducers in practical applications. The aim of this paper is to prove this, i.e., to prove in real experimental conditions that a FOS system achieves the same sensitivity to the acoustic emission as the standard PZT system.

Hence, the paper is organized in order to compare the PZT and the FOS sensor under the same experimental conditions. The AE events are generated by considering the case of fatigue crack propagation in a HEM220 carbon steel beam submitted to fatigue cycles. After a description of the FOS schemes, details of the FOS transducer are given. In the second half of the paper, the conventional and novel sensors are applied to the same experimental conditions, and the results are finally discussed.

## 2. The Coherent Fiber Optic Sensor

The detection of acoustic waves in solids emitted during fatigue and crack propagation phenomena dates back to the dawn of the invention of fiber optic sensors. An important paper appeared at the beginning of the 1990s [4], where the authors attempted to develop a fiber optic sensor dedicated not only to the detection but also to the localization of acoustic events. Starting from these years, a large number of contributions appeared in the literature for all the following decades. Over the first decade of the 2000s, the detection and localization of AE events has been assessed in different materials. In [5], an embedded optical-fiber sensor was proven to monitor crack propagation in concrete beams.

The sensing of AEs at a number of selected points on an optical fiber is achieved by the several fiber-optic transducer placed along the sensing fiber. The proposed sensor proved to be effective in localization of AE events, even though the collected signals are of high energy content and amplitude. The sensor sensitivity is not assessed in the paper, which limits the comparison with other systems. In [6], a fiber-optic interferometric sensor is proposed to detect and localize AE events on a steel plate, while a similar approach was adopted in [7] where AE events were studied on an aluminum plate.

In particular, AE have been simulated via steel ball-dropping on a plate, which produces high intensity signals. In [8], a fiber optic sensor for the detection of precursory acoustic emissions in rockfall events was proposed and experimentally characterized. The sensor, based on an interferometric scheme, was compared to a commercial PZT sensor. Another interferometric scheme was adopted in [9], where a fiber-optic based acoustic emission sensor was proposed to detect the vibrations produced by ultrasonic waves propagating in a solid body.

Similarly, in [10], a fiber-optic interferometric sensor was addressed to detect ultracoustic guided waves in pipes. In [11], a novel fiber-optic sensor based on a polarization modulation was presented for AE detection in large structures and compared to PZT transducers.

In more recent years, Fiber Bragg Gratings (FBG)-based techniques have also been investigated: for example, in [12], a FBG-based AE measurement technique was employed to monitor crack activity in civil engineering structures and sites. In [13,14,15], FBG sensors with high sensitivity over broad bandwidths were proposed for AE detection in composite plates. AE generated by crack formation in an aluminum panel was investigated through a FBG-based sensor in [16], while in [17], the performances of a FBG AE sensor on the surface of a thin polymer-bonded explosive (PBX) material were analyzed.

The localization of AE events, in addition to other acoustic events due to fretting within joints, was also demonstrated in [18] with a multiplexed array of three sensors. Even though these results are promising and, for the first time, a fiber-optic spatial resolution was investigated in AE sensing, the layout presented comprises a distributed-fiber laser sensor, which contributes to creating a complex and expensive solution, which suffers from directional sensitivity of the measurand. This angle-dependency is one of the limiting intrinsic factors correlated to FBG-based solutions.

One of the most recent contributions, presented by the authors in a previous work [19], analyzed a novel fiber-optic based solution for impact detection on an aluminum plate. In this case, the sensor showed promising resulta when compared with commercial PZT sensors. In the following year, in [20], another fiber-optic sensor wAs proposed for ultrasonic stress wave detection: the optical sensor was used to detect guided wave signals on an aluminum plate, and subsequently it was tested on a reinforced concrete beam.

In this case, as in most of the previous work here presented, the sensitivity of the proposed sensors was not fully assessed. Moreover, the analyzed signals were of high-intensity content. The topical interest of a fiber-optic based alternative for AE sensing was not, heretofore, fully addressed. The aim of the proposed work is to meet the need of a fiber-optic based solution for AE sensing, which can ensure comparable performance with respect to the current state-of-the art PZT sensors in detecting AE events.

When a light beam propagates along an optical fiber, some of its features, such as the propagating wave vector, are modulated according to the external influences, such as mechanical and thermal deformation, acting on the fiber itself [21]. Fiber optic interferometric sensors are designed to measure these external perturbations by looking at light phase changes induced by the propagation of wave vector modulation.

Surface-bonded sensors, such as the one proposed in this research work, read the strain transferred from the structure to the fiber: the strain field produces a fiber-optic perturbation, which, in turn, produces an optical response. The amount of phase changes in phase-based sensors is linearly proportional to the strain field transferred to the fiber, the fiber-optic material properties, and the configuration by which the optical fiber is bonded to the specimen. The optical phase delay ϕ (in radians) of light passing through a fiber is given by [21]:

(1) $$\Delta \phi \left(t\right)=n\beta_{0}\left(1-\frac{n^{2}}{2}\left[p_{12}-\mu \left(p_{11}+p_{12}\right)\right]\right)\int_{0}^{l}\varepsilon_{xx}\left(x,t\right)\;dx,$$

where *n* is the effective optical fiber refractive index, $\beta_{0}$ is the vacuum propagaction wave-vector, $p_{ij}$ are the photo-elastic constant, μ is the Poisson’s ratio of silica, $\varepsilon_{xx}$ is the strain in the optical fiber axis direction, and *l* is the optical fiber sensing length. Equation (1) shows that the optical phase change Δϕ(t) is a function of integral deformation.

In particular, the overall phase modulation Δϕ(t) of the light beam propagating into the sensing fiber is obtained by summing all the infinitesimal phase-change contributions dϕ(x,t) along the length dl of the sensor; for this reason, it can be seen as an integral phase sensor.

Without loss of generality, it can be assumed that $p_{11}=+0.121$, $p_{12}=+0.270$, μ=0.17, n=1.45 [21], and, if the sensor is operating at 1550 nm, Equation (1) becomes simply:

(2) $$\Delta \phi \left(t\right)=4.57\cdot 10^{6}\int_{0}^{l}\varepsilon_{xx}\left(x,t\right)\;dx,$$

which shows the extreme sensitivity that the coherent optical fiber sensor can reach.

**Figure 1:**
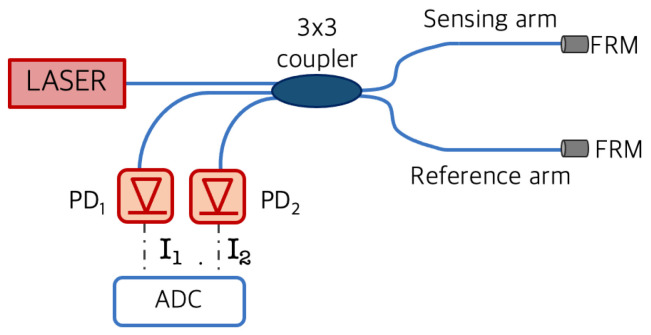
Michelson interferometer layout.

The interferometric fiber optic sensor proposed in this work relies on a coherent detection scheme [22]. The coherent receiver comprises a 3 × 3 coupler in which two ports were chosen as the reference and sensing arm of the interferometer. These ports are terminated with Faraday-Rotator-Mirrors (FRMs), which guarantee the retracing of signal polarizations and, thus, fixed polarizations at the splitter in order to avoid signal fading in interferometric detection schemes [23]. The 3 × 3 coupler performs a passive stable homodyne demodulation where the output signals are $120^{\circ }$ phase shifted and the corresponding receiver photo-currents $I_{1}$ and $I_{2}$ as depicted in the schematic of Figure 1.

As the output of a 3 × 3 coupler, $I_{1}$ and $I_{2}$ can be written as [24]:

(3) $$\begin{array}{c} \begin{array}{c} I_{1}=C+B\cos\left(\Delta \phi \left(t\right)-2/3\pi \right),\\ I_{2}=C+B\cos\left(\Delta \phi \left(t\right)+2/3\pi \right), \end{array} \end{array}$$

where Δϕ(t) is the phase difference induced by the dynamic event between the sensing and reference arms of the interferometer, and *C* and *B* are constants depending on the coupling coefficient of the 3 × 3 coupler.

$I_{1}$ and $I_{2}$ can be digitally sampled by an opportune Analog to Digital Converter (ADC), properly normalized, and processed to retrieved the desired Δϕ(t) through the atan of $\left(I_{1}+I_{2}\right)/\left(I_{1}-I_{2}\right)$ [25]. Due to the adopted coherent demodulation scheme, based on a 3 × 3 coupler, the phase signal Δϕ(t) can be retrieved in a completely passive way without the need for an active feedback for quadrature point stabilization as in conventional fiber optic interferometry.

The minimum phase detection is established by the minimum achievable signals. Considering the spectral content of Acoustic Emission, we decided to adopt low-noise PIN photo-receivers with $B_{el}=200$ kHz electrical bandwidth and Noise Equivalent Power NEP=0.19 pW/$\sqrt{\mathrm{Hz}}$. Since the minimum detectable signal power is:

(4) $$P_{s}^{min}=NEP\sqrt{B_{el}},$$

this resulted in an expected $P_{s}^{min}=0.12$ nW, corresponding to a minimum detectable phase of:

(5) $$\Delta \phi^{min}=\frac{\Delta P_{s}^{min}}{P_{s}^{\pi }},$$

where $P_{s}^{\pi }$ is the power corresponding to a half-fringe excursion, which is equal to 250 μW in our set-up. By introducing this value in Equation (5), the expected $\Delta \phi_{min}\approx 0.5$ μrad can be obtained.

## 3. Fiber Optic Transducer

In acoustic sensing, the fiber optic transducer design is important because it has the task of transferring the ultra-acoustic perturbation of the material in a strain of the optical fiber. The evaluated $\Delta \phi_{min}$ of the previous chapter sets the minimum resolution of the fiber optic interferometer. However, this does not take into account the sensitivity of the fiber-optic transducer, which describes the smallest absolute amount of strain-induced phase change that can be detected by the sensor. In order to increase the sensitivity and to design a fiber-optic transducer able to read small perturbations, one has to increase the fiber sensing length. Intuitively, from Equation (2), it is clear that the longer the fiber length, the higher the integral strain value.

There are two main approaches to lengthen the sensing fiber: by wrapping the fiber in a coil around a solid cylinder (solid cylinder transducer, SCT) or by arranging it in multiple loops (stand alone spiral, SAS). The first approach is the one adopted in [26], in which the fiber was wrapped around an acrylic skeleton and tested for AE sensing with an aluminum plate. This solution offers the possibility to wrap meters of fiber and, thus, to easily increase the sensing fiber length; however, since this type of transducer does read the strain induced by the acoustic wave traveling in the cylinder, it suffers from energy dissipation due to the interface between the cylinder and the metallic plate under test.

In the second configuration instead, the fiber optic is wound like a spiral, and this latter is set at $90^{\circ }$ with respect to the metal surface in order to be adhesively bonded directly onto the structure. This solution was investigated by the authors in a previous work [19], in which this layout showed promising results in impact detection on an aluminum plate. Regarding this configuration, the advantages include the direct detection of waves traveling in the metal and the possibility to increase the sensitivity by adding more loops to the ring, which, however, could compromise the sensor punctuality in the case of a high number of loops.

In order to properly design the fiber-optic transducer, two layouts were compared: the SCT and the SAS were directly compared by varying only the geometry while maintaining the same length of sensing fiber. By using a cylinder of 10 mm in height and a 10 mm radius, it is possible to wrap as much as 30 fiber loops in total for both configurations. However, while, in the first configuration, a solid quartz cylinder was adopted to better match the acoustic wave impedance at the interface between metal and cylinder, in the latter, a 3D printed polymeric ring was chosen to lighten the supporting structure.

**Figure 2:**
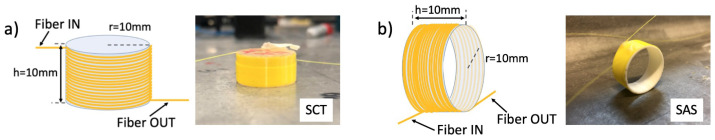
Fiber optic ultrasonic transducer (**a**) quartz cylinder (SCT) and (**b**) 3D-printed ring (SAS).

The detailed layout for both configurations is depicted in Figure 2. Since both configurations present a sensitivity that is a function also of the bonded fiber section, 5 mm was selected as the sensing fiber length, being a good compromise between sensitivity and resolution, as will be discussed later in this section.

**Figure 3:**
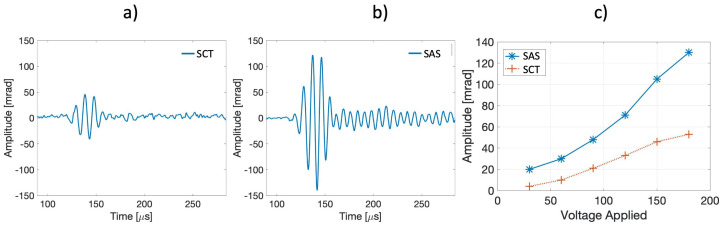
The recovered signal in the case of the (**a**) SCT configuration and (**b**) SAS configuration, (**c**) comparison between the detected amplitude as a function of different five-cycle toneburst amplitudes.

The sensitivity comparison was investigated by generating an AE signal and comparing the amplitude of the collected signals of the two configurations. The cylinder and the spiral were placed on a metal plate at a 100-mm distance from a PZT actuator. The AE signals were obtained by driving a PZT actuator with a five-cycle toneburst, shaped with a Hamming window, at 100 kHz. The results are shown in Figure 3. In particular, in Figure 3a,b, an example of the collected signal is reported, in the case of 180 V applied to the PZT actuator for the SCT and the SAS configurations. Both detected signals in Figure 3a,b refer to the same anti-symmetric A0 wave mode propagating in the metal plate as the respective Time-of-Arrivals of the five-cycle toneburst.

Several experiments were performed by sweeping the voltage applied to the PZT actuator, and the results are reported in Figure 3c. The spiral configuration overdoes the cylinder performance: it is evident that the sensitivity obtained by the SCT was significantly lower than with the SAS. In the authors’ opinions, this difference can be ascribed to the transducing process: in the SCT, the optical fiber wrapped read the cylinder deformations, which means that the acoustic wave energy had to be sufficient to strain the significant cylinder mass; on the contrary, in the SAS, the optical fiber read the acoustic wave strain directly.

To conclude, the SAS configuration appeared as the most promising in detecting AE signals.

As stated in [19], a further key parameter affecting the FOS response was the ratio between the ultrasonic wavelength and the actual fiber length bonded to the structure. In fact, the sensor performed a spatial integration of the ultrasonic signals over the entire sensing length: if an excessive sensing length was chosen, the overall integration drastically degraded the sensor sensitivity.

On the other hand, if the number of loops is increased, the signal bending losses can become too high, and the sensor results may not be punctual, leading to an inaccurate time of arrival evaluation. An optimal choice in terms of the number of loops and bonded sensing fiber length, thus, resulted in a coil of 30 loops, each with a 5-mm bonded section, which guaranteed a good sensitivity in the whole acoustic wavelength range of interest of [18–110] mm.

## 4. Preliminary Tests: Pencil Lead Break

**Figure 4:**
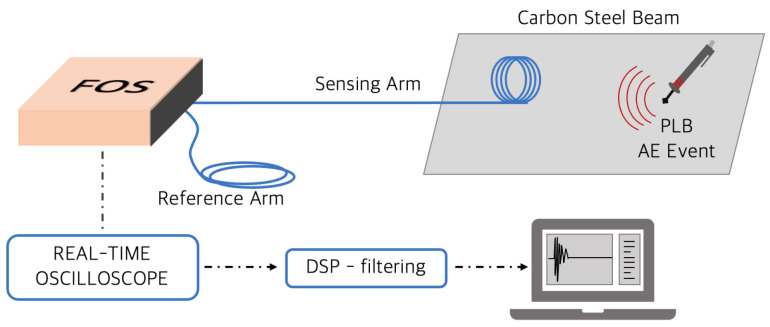
Experimental layout of the FOS sensor.

The experimental set up is shown in Figure 4. Only the fiber-optic sensor transducer is bonded on the metallic structure under test, which is the sensing arm of the MI, while the reference arm is kept close to the sensor interrogator. The signals coming from the photoreceivers are sampled by a 10 MS/s real-time oscilloscope board from Picoscope model 5442D, with 16 resolution bits and a dynamic range of ±0.2 V. These characteristics guarantee an effective overall noise of about 20μrad on the reconstructed and filtered phase. This noise contribution, thus, represented the actual noise level of the overall FOS detection system.

**Figure 5:**
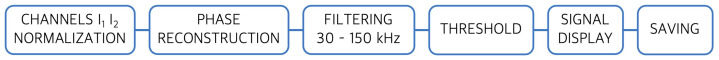
LabVIEW SW steps schematic implemented for the FOS demodulation algorithm.

To recover the phase information Δϕ(t), signal $I_{1}$ and $I_{2}$ from the photodetectors were normalized and demodulated in real-time with the algorithm described in [25] developed in LabVIEW. For the AE signals in the band of [30–150] kHz, a digital band-pass filter was also applied on the recovered phase to reduce the environmental noise contribution. The acquisition algorithm implemented in LabVIEW is schematized in Figure 5.

**Figure 6:**
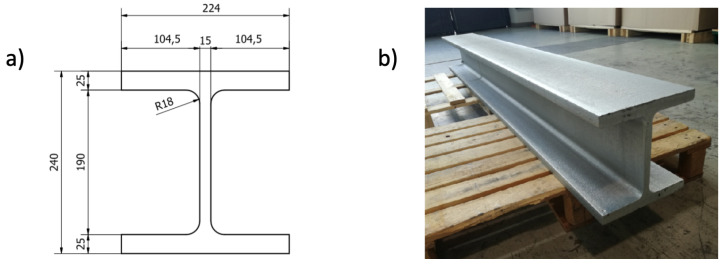
H-Shaped HEM220 beam: (**a**) sketch, (**b**) picture.

The metallic structure under test is a 15-mm thick, 2-m long, and zinc-coated HEM220 beam (EN 10025:1990/A1:1993) made of S355 carbon steel. Its sketch and picture are reported in Figure 6. In this kind of slender and plate-like structures, AE events generate elastic waves propagating in terms of guided waves, i.e., multimodal (dispersive) waves whose number of excited modes depends on the excitation frequency. For most practical purposes, only the fundamental symmetric (compressive) S0 and anti-symmetric (flexural) A0 wave modes are used.

These fundamental wave modes are independent of each other and propagate at different velocities, depending on the thickness-frequency product, based on their relative dispersion curves [27]. Group and phase velocities values were calculated by Vallen AE-Suite Software R2017.0504.1, according to the parameters of the metallic structure. The chosen features for the simulations were the Young modulus E= 210,000 MPa, Poisson ratio ν=0.30, and density ρ=7.85 kg/dm${}^{3}$.

**Figure 7:**
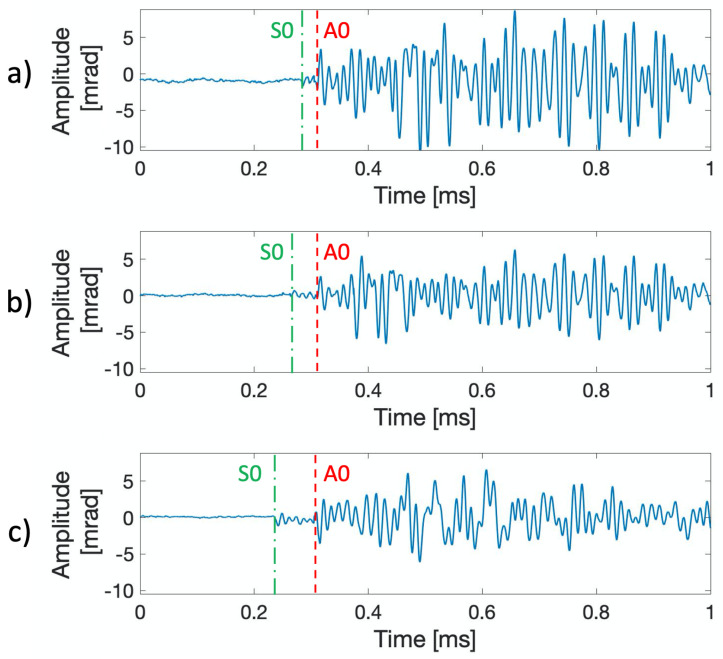
Detail of the recovered signals of FOS during PLB tests on the HEM220 beam: (**a**) 100 mm distance, (**b**) 200 mm distance, and (**c**) 300 mm distance.

Before performing AE detection during a fatigue crack growth test, the proposed FOS was tested to detect guided waves generated by Pencil Lead Break (PLB) tests, also known as the Hsu–Nielsen source. The Hsu–Nielsen source is an artificial method of generating AE signals, which can roughly represent an acoustic emission damage source [28]. PLB tests have been conducted by increasing the distance between the spiral fiber and the PLB breaking point. As reported in Figure 7, PLB tests were performed at 100, 200, and 300 mm on the carbon steel beam.

Both the S0 and the A0 times of arrival could be identified, even at the greatest distance. The FOS was able to detect signals up to 300 mm distance with a good Signal-to-Noise Ratio (SNR), which also allowed the detection of the S0 wave packets, which had a lower energy content with respect to the A0 waves. The Δt between the S0 and the A0 times of arrival are in agreement with the theoretically evaluated wave group velocities for the carbon steel of Table 1. In particular, the theoretical $c_{g}\left(S0\right)=5011$ m/s and $c_{g}\left(A0\right)=3090$ m/s were measured at f≈70 kHz as the main frequency of the recorded waves.

## 5. Fatigue Crack Propagation Test

**Figure 8:**
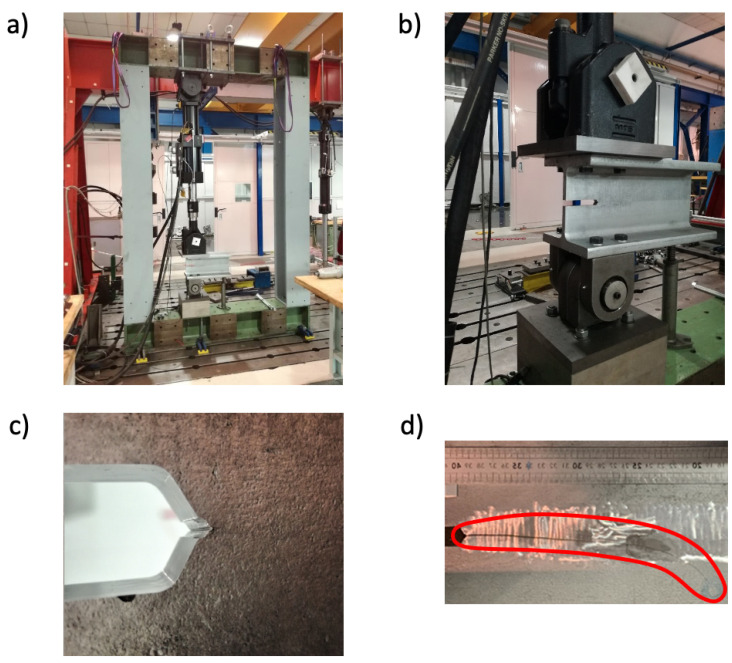
The experimental set up for the fatigue crack propagation test. The experimental set up for the fatigue crack propagation test. (**a**) MTS servo-hydraulic actuator, (**b**) rotational hinges, (**c**) sharp notch at the crack tip and (**d**) final path and morphology of the fatigue crack.

A fatigue crack propagation test was run on a 500 mm long chunk cut from the HEM220 beam described in Section 4. A general view of the experimental set up for the fatigue crack propagation test and of the final result are summarized in Figure 8. In particular, an MTS servo-hydraulic actuator model 244.31, equipped with an MTS load-cell model 661.22D-01 with a maximum nominal load equal to 250 kN and a specifically designed and manufactured structural frame were adopted (Figure 8a) for carrying out the test. More details are described in the following.

In order to induce crack initiation within the desired region of the beam, i.e., its web, and to decrease the crack initiation time, a sharp notch (Figure 8b,c) was manufactured, in the HEM220 beam by mechanical machining (milling) and sharpening through a razor blade. Then, the fatigue crack was initiated and propagated by subjecting the HEM beam to mode-I (opening) loading with respect to the prospective crack plane.

This loading configuration was achieved reproducing the same boundary conditions (Figure 8b, where the presence of the two required rotational hinges is highlighted) characterizing the standardized small-scale fracture mechanics specimen known as “compact tension” [29]. The test was carried out under load-controlled conditions applying a constant amplitude sinusoidal fatigue load with a frequency of 1 Hz, a maximum load level equal to 100 kN, and a minimum one equal to 10 kN, i.e., with a stress ratio R=0.1. Finally, Figure 8d reports the final path and morphology of the fatigue crack at the end of the test.

**Figure 9:**
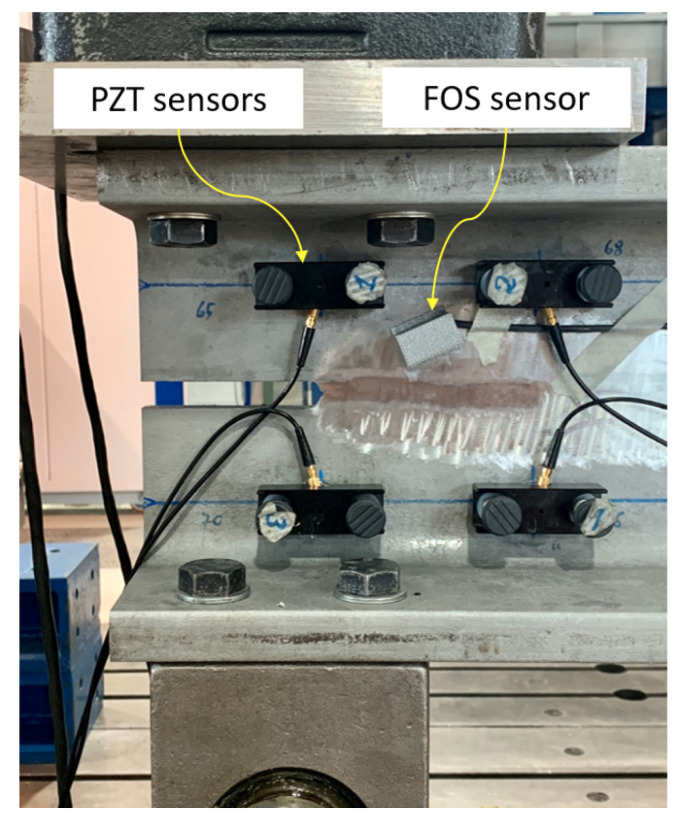
Layout for acoustic emission sensing by PZT sensors and the proposed FOS.

Figure 9 shows the PZT sensors and FOS applied to acquire AE events during the fatigue crack propagation. As can be seen, the former were secured on the structures by means of magnetic holders, while the latter was protected by a 3D-printed case together with a protective tube for the fibers. The complete PZT measurement chain was composed of four Vallen VS150-M piezoelectric sensors, each equipped with a 34-dB Vallen AEP5 pre-amplifier and connected to an eight-channel Vallen ASMY-6 acoustic emission acquisition unit. Moreover, the rules for the acquisition of AE events and for the management of the acquired data were defined using Vallen AE-Suite Software R2017.0504.1.

In particular, the acquisition threshold was set at 50 dB (with respect to a reference voltage amplitude of 1μV) based on the analysis of the background noise (≈40μV) observed during the fatigue test, and a digital passband filter [25–300] kHz was adopted to further skim meaningless signals. The AE events fulfilling the requirements defined by the acquisition threshold and the digital filter were recorded in terms of the traditional parametric features of the AE transient hits (amplitude, energy, duration, counts, and rise time) and in terms of the full waveform sampled at 5 MS/s.

**Figure 10:**
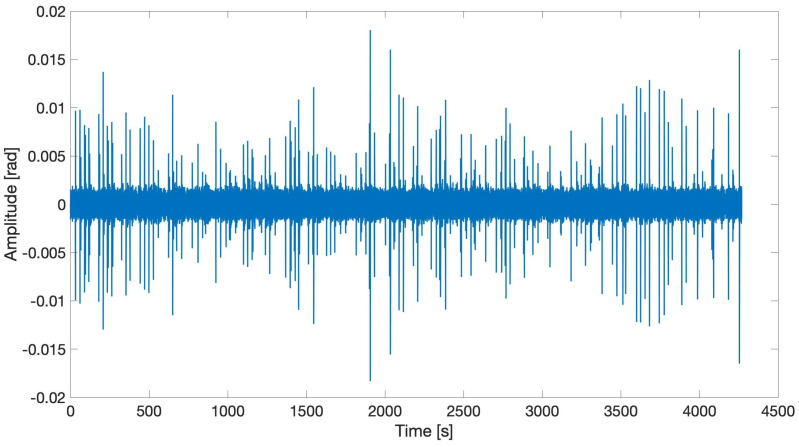
Example of a burst of AE signals detected by the FOS sensor during fatigue crack growth testing.

Figure 10 reports the signals recovered by the FOS sensor when the crack was approximately 100 mm long. AEs cannot be assumed as repeatable sources both in terms of the energy content and time duration, as noticeable from Figure 10. In fact, AE events typically show up as a random burst of pulses produced after a rapid release of energy from localized sources within the material. Therefore, the acquisition SW developed in LabVIEW requires the implementation of a specific threshold on the recovered phase only after filtering to select only the useful signals.

**Figure 11:**
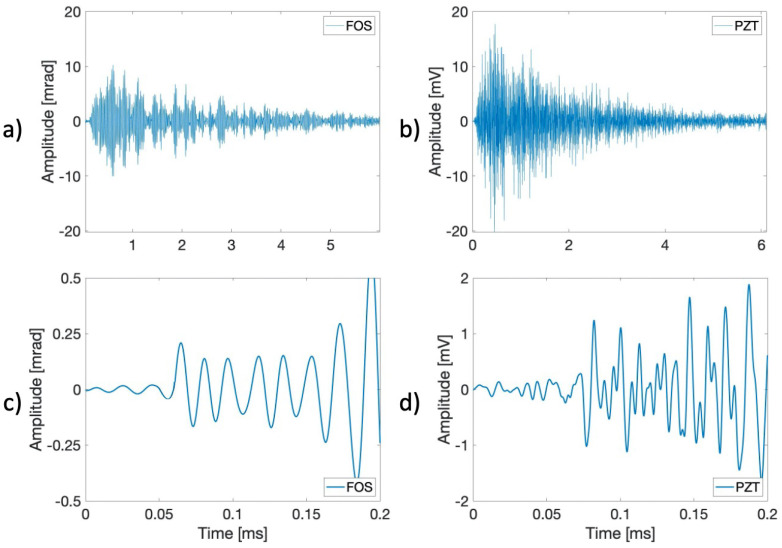
Comparison of the recovered signal during the fatigue growth crack test: (**a**,**c**) FOS-registered AE signal and (**b**,**d**) PZT-registered AE signal.

In Figure 11, the detailed AE signals recovered by the FOS and the PZT sensors are depicted, showing the typical temporal behavior of an AE event registered during a fatigue growth crack test. AE durations can be comparable, while the frequency content is different mainly due to the resonant frequency response of PZT that enhances the higher AE frequencies. To assess and compare the performance of the proposed FOS sensor with respect to PZT, the SNR of the two detected AE signals was evaluated, assumed as the ratio between the average background noise level and the maximum peak amplitude of the AE signal. In particular, the resulting SNRs were $SNR_{FOS}=26.19$ dBV and $SNR_{PZT}=27.88$ dBV, respectively, for the FOS sensor and the PZT sensor.

To further confirm this result, a *z*-test was run over 1000 recovered SNR samples, which were collected during a portion of the fatigue growth crack test. This type of test is suitable to determine whether two population means, that is the two evaluated SNR samples, are different when the variance is known and the number of sample is large. The results reported in Table 2 confirm that the two sensors guaranteed an almost equal SNR mean value and similar confidence interval of 95%, thus, proving comparable performance in AE monitoring. The large confidence interval calculated for both type of sensors is due to the random nature of AE events, which cannot be assumed as repeatable sources.

In addition to SNR assessment, a comparison of the detected energy levels was carried out, showing that PZT and FOS detected signals featuring similar energy peaks in time. The energy was calculated in the same time span of 6 ms as the integral of the modulus square of the signal. This statement holds if the compared signals are of the same AE event. The energy values were evaluated in dB with respect to the reference voltage amplitude of 1μV and phase of 1μrad for PZT and FOS, respectively.

**Figure 12:**
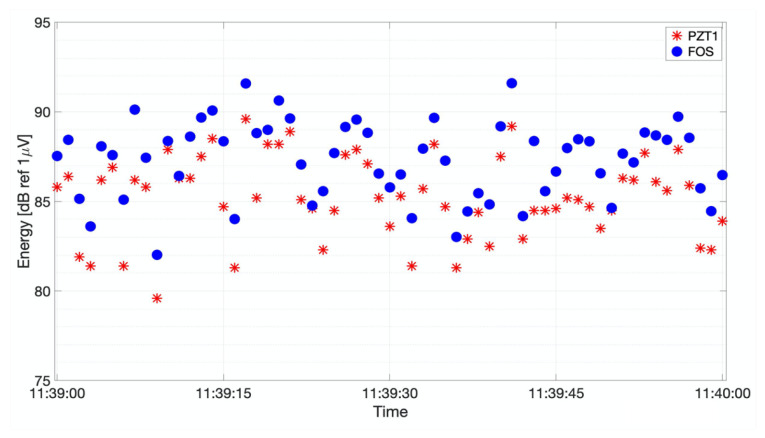
The evaluated energy during 1 min of the fatigue growth crack test at L = 100 mm crack length.

**Figure 13:**
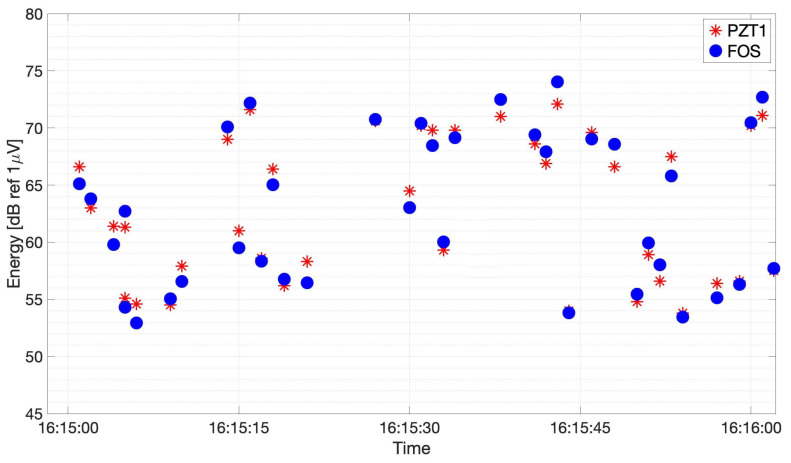
The evaluated energy during 1 min of the fatigue growth crack test at L = 15 mm crack length.

Part of the results from the comparison of the two sensors are reported in Figure 12, where, for the sake of clarity, only 1 min of registered high-energy signal values is detailed. Indeed, similar behavior to that depicted in Figure 12 was observed during the whole fatigue test, though with different energy levels depending on the position of the crack tip along its propagation path. As an example, Figure 13 shows the energy signals recovered when the crack tip was only 15-mm long.

In this case, the peak energy values were much lower with respect to those in Figure 12. Indeed, during the initial part of its propagation, the crack was typically not very energetic, while, after about 100 mm, in correspondence with the area where the crack began to deviate from its straight path, the emissions started to be highly energetic.

To further confirm this result, a *z*-test was performed on the evaluated energies from the two sensors. Table 3 reports the evaluated mean value μ and confidence interval of 95%, which gave α=0.05, for the FOS and the PZT, calculated over 1000 recovered energy samples, collected during the fatigue growth crack test when the crack length was both 15 and 100 mm. In spite of the resulting large confidence interval due to the AE events random nature, a good agreement was found between FOS and PZT, both in terms of the mean energy value and confidence interval, thus, confirming that the two sensors collected similar energy values.

For the 100-mm case, the mean value difference of 3.31 dB might be ascribed to the fact that the FOS sensor was, in this case, closer, with respect to PZT1, to the crack tip as shown in Figure 9, and thus the A0 wave modes arrived less attenuated. For the 15 mm, the difference was lower as the FOS and PZT1 sensors were at similar distance from the crack tip. The two confidence intervals, on the contrary, were almost equal, confirming that the two sensors collected similar signal energies. The large confidence interval calculated for both types of sensor endorses the random nature of AE events, which cannot be assumed as repeatable sources.

## 6. Summary and Comments

In this paper, a novel fiber optic AE sensor with detection characteristics comparable with the conventional PZT sensor was presented. Two configurations were taken into consideration for the fiber-optic transducer—a solid cylindrical structure (SCT) and a stand-alone spiral (SAS)—and the latter proved to have the highest sensitivity in detecting ultracoustic signals.

The SAS configuration was chosen as a suitable FOS transducer, and thus it was further tested with PLB preliminary measurements and demonstrated a good SNR in detecting symmetric and anti-symmetric wave modes propagating in a HEM220 carbon steel beam. The time of arrivals of the two wave modes were theoretically calculated using Vallen AE-Suite Software according to the parameters of the metallic structure and were compared with the evaluated time of arrivals. Good agreement was found in the comparison of the theoretical and evaluated time of arrivals.

The FOS performance was compared to the standard PZT AE sensor that is commercially available in crack monitoring during a fatigue growth crack test, run on a 500-mm long chunk cut of a carbon steel beam. A direct relationship between PZT and FOS was found: recovered signals of the same event showed similar time durations and a comparable SNR, even though the two sensors had different frequency responses. To better compare them, an energy analysis of the detected event was performed: a good agreement was found between the PZT and FOS pulse energy behavior.

To conclude, the novel FOS sensor exhibited performances comparable to the standard commercially available PZT when tested during fatigue crack growth tests, thus, proving to be a valid alternative to PZT in AE sensing while holding all the advantages of being fiber-optic-based with a competitive cost of about half of the current PZT transducer control units. The authors believe that these early results may open up remarkable prospects for applying fiber optic sensors in AE detection. This optical fiber transducer is very simple to manufacture and can be easily multiplexed, thus, forming the basis for the construction of complex sensor networks that can be remotely installed in field applications.

## Figures and Tables

**Table 1:** The theoretical and measured Δt between the S0 and A0 times of arrival, for the 100, 200, and 300 mm distances.

L [mm]	$c_{g}\left(S0\right)$ [m/s]	$c_{g}\left(A0\right)$ [m/s]	$\Delta t_{\mathrm{TH}}\;$[μs]	$\Delta t_{\mathrm{EVAL}}\;$[μs]
100	5011	3090	12.4	14.6
200	5011	3090	24.8	28.0
300	5011	3090	37.3	40.2

**Table 2:** The mean value and critical value at 95% for FOS- and PZT-collected SNRs.

Sensor	Mean Value μ [dB]	Confidence Interval at 95% [dB]
FOS	24.68	8.13
PZT	25.95	7.40

**Table 3:** The mean value and critical value at 95% for the FOS- and PZT-collected energies.

Crack Length	Sensor	Mean Value μ [dB]	Confidence Interval at 95% [dB]
15 mm	FOS	64.26	13.10
	PZT	65.72	14.2
100 mm	FOS	79.36	20.57
	PZT	76.05	20.43

## Data Availability

Not applicable.
